# Dr. Alonzo Pettit

**Published:** 1908-12

**Authors:** 


DR. ALONZO PETTIT, of Elizabeth, N. J., died at his home, November 14, 1908, from disease of the heart, aged 66 years. He helped to organise the Elizabeth General Hospital and served as a member of the consulting staff for many years. He graduated at the University of Buffalo in 1867.

